# Relationships between knee muscle strength and functional performance in professional soccer players

**DOI:** 10.3389/fspor.2026.1832191

**Published:** 2026-06-17

**Authors:** Tamiris Beppler Martins, Raphael Schmidt de Mesquita, Thiago da Silva de Jesus, Guilherme Fleury Badin, Lucas Fernando Correia Gomes, Gabriel Golle Eitelwein, Rodrigo Okubo

**Affiliations:** 1 Departamento de Fisioterapia, Centro de Ciências, Tecnologias e Saúde, Universidade Federal de Santa Catarina, Araranguá, Santa Catarina, Brazil; 2 Programa de Pós-graduação em Fisioterapia, Universidade do Estado de Santa Catarina, Florianópolis, Santa Catarina, Brazil; 3 Departamento de Fisioterapia, Centro de Ciências da Saúde e do Esporte, Universidade do Estado de Santa Catarina, Florianópolis, Santa Catarina, Brazil

**Keywords:** hop test, isokinetic dynamometry, limb symmetry index, modified star excursion balance test, vertical jump

## Abstract

**Introduction:**

Functional tests such as the Modified Star Excursion Balance Test (mSEBT), hop tests, and jump tests are widely used to monitor performance, neuromuscular function, and readiness in soccer players; however, their relationship with knee muscle strength remains unclear.

**Methods:**

This cross-sectional study investigated the associations between commonly used functional test outcomes and isokinetic quadriceps and hamstrings peak torque at 60°/s in professional male soccer players. Forty asymptomatic athletes completed the functional test battery, and isokinetic data were available for 38 players. The assessment included the mSEBT, single-leg hop for distance, bilateral hop, bilateral vertical jump, and single-leg vertical jump. Concentric isokinetic peak torque of the quadriceps and hamstrings was assessed at 60°/s using a Biodex System 4 dynamometer. Limb Symmetry Indices were calculated for strength and functional measures.

**Results:**

Bilateral vertical jump height showed moderate correlations with quadriceps peak torque, whereas single-leg vertical jump performance showed weak, side-specific associations with right hamstrings torque and left quadriceps torque. No meaningful correlations were found between single-leg hop performance and knee muscle strength. Strong inter-limb associations were observed for the mSEBT and single-leg hop test, but these tests were not significantly associated with one another. Functional symmetry indices were high, although they were not associated with quadriceps strength symmetry.

**Discussion:**

These findings indicate that functional tests reflect distinct neuromuscular attributes and should not be considered interchangeable with isolated measures of knee muscle strength. Therefore, return-to-sport and performance-monitoring decisions should integrate task-specific functional testing with objective strength assessment.

## Introduction

Functional assessment plays an important role in sports physical therapy by supporting performance monitoring, identifying neuromuscular deficits, and informing injury prevention and return-to-sport decision-making in athletes exposed to high physical demands (1). Among the tools most commonly used in soccer, the Modified Star Excursion Balance Test (mSEBT), hop tests, and vertical jump tasks are applied to quantify dynamic balance, unilateral control, and explosive performance (2, 3). However, the extent to which these field-based outcomes reflect isolated knee muscle capacity remains uncertain, particularly when compared with torque production obtained under standardized laboratory conditions (4, 5).

In both clinical and sports settings, functional tests are often interpreted as indirect markers of muscular performance, yet their underlying demands differ substantially. The mSEBT depends heavily on postural control, hip and trunk stabilization, ankle-foot control, and sensorimotor integration during a constrained reach task. Hop and jump tests require rapid force generation, stretch-shortening cycle utilization, intersegmental coordination, and force transmission across the hip-knee-ankle chain during take-off and landing. By contrast, isokinetic dynamometry isolates knee extensor and flexor torque under controlled angular velocity and range-of-motion conditions (6, 7). By contrast, isokinetic dynamometry isolates knee extensor and flexor torque under controlled angular velocity and range-of-motion conditions. These biomechanical differences suggest that functional tests may relate to strength to different degrees rather than acting as interchangeable proxies.

Previous evidence is also uneven across outcomes. Associations between knee extensor strength and vertical jump performance have been reported with some consistency, whereas findings for hop-based tasks and dynamic balance measures are more heterogeneous and, in some contexts, absent. This inconsistency may reflect differences in sample characteristics, competitive level, protocol design, outcome definitions, and the relative contribution of proximal control, distal stiffness, and landing strategy to each task (8, 9). In professional soccer players, clarifying these relationships is particularly relevant because performance monitoring and return-to-sport decisions frequently combine strength testing with field-based functional tests. A better understanding of where measures converge and where they diverge may improve clinical interpretation and test selection.

In addition to absolute performance, inter-limb symmetry is frequently used as a clinical criterion in rehabilitation and return-to-sport contexts. However, symmetry in functional performance does not necessarily imply symmetry in isolated muscle strength, since different tests may be influenced by compensatory movement strategies, coordination patterns, and task-specific demands. Examining the relationship between functional and strength-based symmetry indices may therefore provide further insight into whether these measures offer overlapping or complementary information.

Therefore, this study aimed to evaluate the relationships between performance in functional tests, including the mSEBT, single-leg hop test, bilateral hop, and vertical jump tasks, and quadriceps and hamstrings isokinetic peak torque at 60 °/s in professional male soccer players. We hypothesized that: (1) vertical jump performance would show moderate positive associations with quadriceps peak torque at 60 °/s; (2) hop and mSEBT performance would show weak or no associations with knee torque because of their greater dependence on task-specific neuromuscular and coordinative demands; and (3) functional symmetry indices would not necessarily be associated with strength symmetry.

## Methods

This cross-sectional study was conducted at the Center for Health and Sports Education of the State University of Santa Catarina (UDESC), Brazil. The study followed the Strengthening the Reporting of Observational Studies in Epidemiology (STROBE) guidelines for observational research. The protocol was approved by the UDESC Human Research Ethics Committee (CAAE: 89354225.8.0000.0118; approval number 7.712.393) and was conducted in accordance with the Declaration of Helsinki. All participants provided written informed consent prior to participation.

### Participants

A convenience sample of professional male soccer players was recruited from a competitive team environment. Athletes were regularly engaged in structured team training and competition at the professional level. Inclusion criteria were: (i) active participation in team training and competition; (ii) absence of lower-limb injury in the previous six months; and (iii) ability to perform all tests without pain. Exclusion criteria were: (i) current lower-limb pain during training or matches, even if not causing time loss; (ii) previous lower-limb surgery; and (iii) any neurological, vestibular, or musculoskeletal condition that could affect test performance. Demographic and sport-related data were collected, including age, height, body mass, field position, preferred kicking leg, and current or previous injury history, defined as any musculoskeletal complaint resulting in absence from training or matches. For study purposes, injury status was determined from structured self-report of current symptoms and previous time-loss injuries, together with pain-free execution of the test battery on the assessment day. All assessments were performed by two experienced physical therapists familiar with the testing protocol. Participants received standardized verbal instructions before each test and were allowed to withdraw at any point in case of discomfort or pain. Preferred kicking leg was self-reported as the leg usually used to kick the ball and was recorded for descriptive purposes and to standardize the order of unilateral tests; it was not used as an analytical factor.

### Procedures

#### Warm-up and testing sequence

All evaluations were performed in a single session under standardized laboratory conditions. Participants completed a 5-min warm-up on a cycle ergometer at 60 ± 5 rpm with a workload of approximately 1 W/kg. Functional performance tests were conducted before isokinetic strength testing to minimize the influence of fatigue on task execution. A rest interval of 90–120 s was provided between maximal trials and test conditions. The full protocol lasted approximately 60 min. Athletes were assessed during the preseason or early competitive phase, and recent training load in the 48 h preceding testing was checked verbally to reduce the likelihood of acute fatigue influencing performance.

#### Modified star excursion balance test (mSEBT)

Dynamic balance was assessed using the mSEBT in three directions: anterior (ANT), posteromedial (PM), and posterolateral (PL). Three tape measures were fixed to the floor in a Y-shaped configuration, with the anterior direction positioned relative to the posteromedial and posterolateral directions as previously described (33). Athletes performed the test barefoot, maintaining single-leg stance on the test limb while reaching as far as possible with the contralateral limb and keeping both hands on the iliac crests. Four familiarization trials per direction were followed by three recorded valid trials. A trial was considered valid when the stance foot remained stable, the contralateral foot did not provide support on the floor, and hand position was maintained throughout the movement (10). Reach distance was recorded at the hallux and normalized to limb length by dividing reach distance by limb length. Limb length was measured in the supine position from the anterior superior iliac spine to the distal tip of the medial malleolus. A composite score was calculated as the mean of the normalized ANT, PM, and PL reach distances for each limb.

### Vertical jump and single-Leg vertical jump

Explosive vertical performance was assessed using the My Jump® smartphone application, which has been validated for estimating jump height from flight time (11). The smartphone camera was positioned approximately five meters from the sagittal plane of motion. Participants performed one familiarization trial followed by three maximal countermovement jumps with hands on the waist in both bilateral and single-leg conditions. The highest valid jump in each condition was retained for analysis. Trials were repeated if the athlete lost balance or used arm swing.

#### Single-leg and bilateral hop tests

Horizontal performance and lower-limb coordination were assessed using single-leg and bilateral hop-for-distance tests (12). In the bilateral condition, athletes performed three maximal forward hops using both lower limbs simultaneously. In the single-leg condition, participants performed maximal forward hops using one limb at a time, starting with the toes positioned immediately behind the marked starting line. Hops were performed wearing sport shoes to better reflect sport-specific conditions. Distance was recorded using a floor-mounted measuring tape. A trial was considered valid when the athlete landed in a stable manner, maintained balance for at least 3 s, and did not perform extra corrective steps. The same verbal instructions were used across limbs and trials. The longest valid attempt in each condition was used for analysis.

#### Isokinetic strength assessment

Knee extensor and flexor torque were measured using a Biodex™ System 4 Pro isokinetic dynamometer (Biodex Medical Systems, Shirley, NY, USA). Participants were positioned seated with the hip at approximately 90° of flexion, the lateral femoral epicondyle aligned with the dynamometer axis of rotation, and the trunk, pelvis, and tested thigh stabilized with straps. Testing was performed concentrically at 60 °/s over a 100° range of motion, corresponding approximately to 90° to 10° of knee flexion. Participants completed five submaximal repetitions for familiarization, followed by a 120-s rest interval and then five maximal repetitions of knee extension and flexion for each limb. Standardized verbal encouragement was provided throughout the test. The highest peak torque value obtained for each muscle group and limb was retained for analysis. The angular velocity of 60 °/s was selected to emphasize maximal concentric torque production under controlled mechanical conditions, whereas the 100° range of motion was chosen to reduce end-range mechanical stress while still encompassing functionally relevant knee angles for soccer-related actions.

#### Limb symmetry index

The Limb Symmetry Index (LSI) was calculated for quadriceps torque, single-leg hop performance, and single-leg vertical jump performance as follows: LSI = lower-performing limb/higher-performing limb (13). This same formula was applied across outcomes, independent of limb dominance, so that values approached 1.0 as symmetry increased. Consistent with commonly used clinical thresholds, values equal to or above 0.90 were interpreted as symmetrical (14), whereas values below 0.90 were interpreted as indicating potential asymmetry (15). Symmetry analyses were prespecified for quadriceps torque, single-leg hop performance, and single-leg vertical jump performance because these measures are commonly used in rehabilitation and return-to-sport monitoring.

### Statistical analysis

Data were analyzed using the Statistical Package for the Social Sciences (SPSS®), version 20.0 (IBM Corp., Armonk, NY, USA). Given the exploratory aim of determining whether commonly used functional measures covary with isolated knee torque, the primary analyses focused on pairwise associations rather than predictive modelling. Multivariable regression was not pursued because the torque subsample was relatively small (*n* = 38), which would increase the risk of unstable estimates and overfitting in models containing multiple correlated functional predictors. Descriptive statistics, including mean, standard deviation, and minimum–maximum values, were used to characterize the sample and summarize performance in the mSEBT, single-leg hop, bilateral hop, vertical jump, single-leg vertical jump, and isokinetic torque variables. Forty athletes completed the functional test battery. Because complete isokinetic data were not available for all participants, analyses involving torque variables were conducted using available-case analysis; accordingly, functional-only correlations were based on *n* = 40, whereas torque-related analyses were based on *n* = 38. Data distribution was examined using the Shapiro–Wilk test. Pearson's correlation coefficient was used for normally distributed variables, whereas Spearman's rank correlation coefficient was used when normality assumptions were not met. Correlation magnitude was interpreted descriptively, exact *p* values were reported, and 95% confidence intervals (CIs) are now provided for the main correlation coefficients. Correlations were explored at two analytical levels: (i) bilateral-level, in which bilateral functional outcomes were compared with torque values from both limbs; and (ii) limb-specific, in which unilateral functional outcomes were matched to torque values from the corresponding limb. Associations among symmetry indices were also examined. Because these analyses were hypothesis-driven and exploratory, no formal multiplicity correction was applied; instead, effect sizes and exact *p* values were emphasized to support cautious interpretation. All analyses adopted a two-tailed approach with *α* = 0.05.

## Results

### Sample characterization

A total of 40 professional male soccer players participated in the study. Mean age was 26.3 ± 5.8 years (range: 18.0–39.4), mean body mass was 79.6 ± 8.7 kg, and mean height was 1.81 ± 0.07 m. Most athletes were evaluated during preseason reintroduction (72.5%; *n* = 29), whereas 27.5% (*n* = 11) were tested during the transfer window. Field positions were distributed as follows: defensive midfielders and center-backs (20.0% each), attacking midfielders and wingers (15.0% each), goalkeepers (12.5%), full-backs (10.0%), and forwards (7.5%). Regarding preferred kicking leg, 72.5% of the players were right-leg dominant.

### Functional performance and isokinetic strength

Group means for functional tests and isokinetic torque are summarized in Table 1. All functional variables were available for *n* = 40 athletes, whereas complete isokinetic torque data were available for *n* = 38. The normalized composite mSEBT score was 0.893 ± 0.038 for the right limb and 0.893 ± 0.041 for the left limb. Bilateral vertical jump height was 42.1 ± 4.1 cm, whereas single-leg vertical jump height was 22.7 ± 3.6 cm for the right limb and 22.6 ± 3.4 cm for the left limb. The single-leg hop for distance averaged 217.9 ± 16.5 cm on the right and 219.0 ± 16.6 cm on the left, while bilateral hop distance was 252.1 ± 19.7 cm. Mean peak isokinetic torque was 308.6 ± 45.4 Nm for the right quadriceps, 302.3 ± 42.0 Nm for the left quadriceps, 203.9 ± 35.5 Nm for the right hamstrings, and 199.6 ± 34.7 Nm for the left hamstrings (Table 1).

**Table 1:** Functional performance and isokinetic peak torque values (mean ± SD; min–max).

Variable^*X*^ ^±^ ^SD [Min–Max]^	Value
Composite mSEBT—Right	0.893 ± 0.038 [0.81–0.96]
Composite mSEBT—Left	0.893 ± 0.041 [0.80–0.96]
Vertical jump (cm)	42.1 ± 4.1 [34.0–52.0]
Single-leg jump—Right (cm)	22.7 ± 3.6 [13.0–30.8]
Single-leg jump—Left (cm)	22.6 ± 3.4 [15.8–29.7]
SLHT—Right (cm)	217.9 ± 16.5 [184.0–250.0]
SLHT*—*Left (cm)	219.0 ± 16.6 [186.0–248.0]
Bilateral hop (cm)	252.1 ± 19.7 [205.0–290.0]
Right quadriceps muscle (Nm)	308.6 ± 45.4 [223.0–407.0]
Left quadriceps muscle (Nm)	302.3 ± 42.0 [201.0–381.0]
Right hamstring muscle (Nm)	203.9 ± 35.5 [111.0–266.0]
Left hamstring muscle (Nm)	199.6 ± 34.7 [121.0–310.0]

SD, standard deviation; Min, minimum; Max, maximum; mSEBT, modified Star Excursion Balance Test; SLHT, Single-Leg Hop Test. Functional variables were available for *n* = 40 athletes, whereas isokinetic torque variables were available for *n* = 38.

### Correlation between functional tests

Strong inter-limb associations were observed within the same functional test. Specifically, single-leg hop performance showed a strong correlation between limbs (*r* = 0.917; 95% CI: 0.848–0.956; *n* = 40; *p* < 0.001), whereas the mSEBT showed a moderate-to-strong correlation between limbs (*r* = 0.789; 95% CI: 0.633–0.883; *n* = 40; *p* < 0.001). No significant correlations were found between mSEBT and single-leg hop performance for either limb (r range: −0.008 to 0.223; *n* = 40; *p* > 0.05), suggesting that these tests capture distinct aspects of lower-limb function. Detailed coefficients are presented in Table 2.

**Table 2:** Correlations among mSEBT and single-Leg Hop test performances.

Variable	mSEBT R	mSEBT L	SLHT R	SLHT L
mSEBT R	1	—	—	—
mSEBT L	0.789* [0.633 to 0.883]	1	—	—
SLHT R	0.158 [−0.161 to 0.447]	0.223 [−0.095 to 0.500]	1	—
SLHT L	−0.008 [−0.319 to 0.304]	0.065 [−0.252 to 0.369]	0.917* [0.848 to 0.956]	1

mSEBT, modified Star Excursion Balance Test; SLHT, Single-Leg Hop Test; R, right; L, left. Values are Pearson's correlation coefficients (r) with 95% confidence intervals in brackets. All correlations in this table were based on *n* = 40.

* *p* < 0.001.

### Symmetry indices

The Limb Symmetry Indices (LSI) for quadriceps torque, single-leg hop performance, and single-leg jump performance showed mean values close to 1.0, indicating predominantly symmetrical performance between limbs. Mean LSI values were 0.92 for quadriceps torque, 0.98 for hop performance, and 0.92 for single-leg jump performance. Descriptive data are presented in Table 3.

**Table 3:** Descriptive statistics of symmetry indices.

Variable	Mean ± SD	Min–Max	95% CI
SYMMETRY_Q	0.92 ± 0.06	0.69–1.00	0.90–0.94
SYMMETRY_HOP	0.98 ± 0.02	0.90–1.00	0.97–0.99
SYMMETRY_JUMP	0.92 ± 0.07	0.67–1.00	0.90–0.94

SD, standard deviation; Min, minimum; Max, maximum; 95% CI, 95% confidence interval; SYMMETRY_Q, quadriceps strength symmetry index; SYMMETRY_HOP, Single-Leg Hop Test symmetry index; SYMMETRY_JUMP, single-leg vertical jump symmetry index. SYMMETRY_Q was based on *n* = 38, whereas the functional symmetry indices were based on *n* = 40.

A weak positive correlation was observed between SYMMETRY_JUMP and SYMMETRY_HOP (*r* = 0.323; 95% CI: 0.013–0.577; *n* = 40; *p* = 0.042). No significant correlations were found between SYMMETRY_Q and SYMMETRY_JUMP (*r* = 0.064; *p* = 0.701) or between SYMMETRY_Q and SYMMETRY_HOP (*r* = −0.118; *p* = 0.482), as shown in Table 4. These findings suggest that symmetry in functional performance does not necessarily reflect symmetry in knee extensor strength. To remain aligned with the original analytical plan, symmetry was interpreted as a continuous construct rather than by adding a *post hoc* categorical threshold analysis.

**Table 4:** Correlations among symmetry indices.

Variable	SYMMETRY_JUMP	SYMMETRY_HOP	SYMMETRY_Q
SYMMETRY_JUMP	1	—	—
SYMMETRY_HOP	0.323* [0.013 to 0.577]	1	—
SYMMETRY_Q	0.064 [−0.261 to 0.376]	−0.118 [−0.422 to 0.210]	1

SYMMETRY_Q, quadriceps strength symmetry index; SYMMETRY_HOP, Single-Leg Hop Test symmetry index; SYMMETRY_JUMP, single-leg vertical jump symmetry index. Values are Pearson's correlation coefficients (*r*) with 95% confidence intervals in brackets. Correlations involving only functional symmetry indices were based on *n* = 40; correlations involving SYMMETRY_Q were based on *n* = 38.

* *p* < 0.05.

## Correlation between power tests and isokinetic peak torque

Task-specific associations were identified between functional performance and knee torque (Table 5). Bilateral vertical jump height showed moderate correlations with quadriceps peak torque for both limbs (right: *r* = 0.485, 95% CI: 0.196–0.697; *n* = 38; *p* = 0.002; left: *r* = 0.405, 95% CI: 0.098–0.642; *n* = 38; *p* = 0.012), whereas no significant correlations were found with hamstring torque. No significant correlations were observed between single-leg hop performance and the corresponding knee torque values. For unilateral vertical jumps, right single-leg jump performance was weakly correlated with right hamstring torque (*r* = 0.346, 95% CI: 0.030–0.599; *n* = 38; *p* = 0.034), whereas left single-leg jump performance was weakly correlated with left quadriceps torque (*r* = 0.326, 95% CI: 0.007–0.585; *n* = 38; *p* = 0.046). No consistent dominance-related pattern was observed across these side-specific analyses, and no other significant associations were found.

**Table 5:** Correlations between functional tests and isokinetic peak torque at 60 °/s.

Variable	Quadriceps muscle—R (*r*/*p*)	Quadriceps muscle—L (*r*/*p*)	Hamstring muscle—R (*r*/*p*)	Hamstring muscle—L (*r*/*p*)
Vertical jump	0.485/0.002* **[0.196 to 0.697]**	0.405/0.012* **[0.098 to 0.642]**	0.208/0.209 [−0.120 to 0.495]	0.185/0.265 [−0.143 to 0.477]
Bilateral Hop	0.030/0.860 [−0.292 to 0.346]	0.047/0.779 [−0.277 to 0.361]	0.080/0.631 [−0.246 to 0.390]	0.069/0.681 [−0.256 to 0.380]
SLHT—R	0.075/0.656 [−0.251 to 0.385]	—	0.136/0.416 [−0.192 to 0.437]	—
SLHT—L	—	0.052/0.756 [−0.272 to 0.366]	—	−0.016/0.926 [−0.334 to 0.305]
Single-leg jump—R	0.288/0.080 [−0.035 to 0.556]	—	0.346/0.034* **[0.030 to 0.599]**	—
Single-leg jump—L	—	0.326/0.046* **[0.007 to 0.585]**	—	0.259/0.117 [−0.066 to 0.534]

R, right; L, left; *r*, correlation coefficient; *p*, *p* value; SLHT, Single-Leg Hop Test. Values are Pearson's correlation coefficients (*r*)/*p*-value with 95% confidence intervals in brackets. All correlations in this table were based on *n* = 38.

Bold values indicate statistically significant correlations (*p* < 0.05).

* *p* < 0.05.

## Discussion

The aim of this study was to examine the relationships between performance in functional tests, including the Modified Star Excursion Balance Test (mSEBT), single-leg hop test, and vertical jump tasks, and isokinetic quadriceps and hamstrings peak torque in professional male soccer players. The main findings were that inter-limb associations within the same functional test were strong, functional symmetry indices were generally high, and the relationships between functional performance and isokinetic torque were modest and task-specific. In addition, symmetry in functional performance was not associated with quadriceps strength symmetry. Together, these findings suggest that commonly used functional tests capture distinct dimensions of lower-limb function and should not be interpreted as interchangeable with isolated measures of knee muscle strength.

### Inter-limb associations and functional symmetry

The strong correlations observed between limbs for the mSEBT (*r* = 0.789) and single-leg hop test (*r* = 0.917) indicate high inter-limb consistency in test performance in this sample of professional soccer players. These findings are consistent with previous reports showing good reproducibility and stable performance in balance and hop-based tasks in athletic populations (16, 17). In the present study, symmetry indices for quadriceps torque, hop performance, and single-leg jump performance were also close to 1.0, indicating that, on average, athletes displayed similar performance between limbs.

However, functional symmetry should not be interpreted as evidence of equivalent underlying muscle function. Although mean symmetry indices were high, no significant associations were observed between quadriceps strength symmetry and the symmetry indices derived from hop and jump tasks. This finding agrees with previous evidence showing that satisfactory functional symmetry may coexist with persistent deficits in isolated knee function, particularly when athletes adopt compensatory strategies to maintain task performance (18, 19). Increased contribution from proximal or distal joints, altered movement coordination, and task-specific motor adaptations may allow athletes to achieve symmetrical functional outcomes despite unresolved strength asymmetries (20). Therefore, symmetry-based interpretation should be contextualized with objective strength assessment, particularly in rehabilitation and return-to-sport settings.

### Vertical jump performance and knee torque

A moderate association was observed between bilateral vertical jump height and quadriceps peak torque in both limbs (*r* = 0.405–0.485), indicating that greater knee extensor torque capacity was related to better vertical jump performance. This result is in line with previous studies showing that quadriceps strength contributes meaningfully, although not exclusively, to countermovement jump performance (21, 22). Mechanistically, the propulsion phase of a vertical jump depends on rapid force transmission across the hip-knee-ankle chain, with a relevant contribution from knee extensor torque to vertical impulse generation. However, this relationship is not expected to be one-to-one because jump height also depends on ankle plantar-flexor behavior, proximal segment coordination, countermovement strategy, and stretch-shortening cycle efficiency (23, 24). In contrast, hop performance relies on coordinated multi-joint contributions, horizontal force orientation, landing control, and limb stiffness regulation, thereby diluting the specific relationship between knee torque and task outcome (25, 26). Consequently, vertical jump testing may serve as a partial but ecologically valid indicator of quadriceps strength, especially when time or equipment constraints limit isokinetic testing. Interestingly, the associations observed for unilateral vertical jumps were weaker and side-specific. The right single-leg jump was weakly associated with right hamstrings torque, whereas the left single-leg jump was weakly associated with left quadriceps torque. Because these coefficients were small and side-specific, they should be interpreted cautiously and not as evidence of a consistent dominance effect. Rather, they reinforce that unilateral jumping performance is influenced by multiple factors beyond isolated knee torque, including balance constraints, segmental alignment, intersegmental timing, and individualized movement strategies. Accordingly, vertical jump tests may provide ecologically relevant information about lower-limb performance, but they should not be assumed to directly represent isolated muscle strength.

### Hop performance and task-specific demands

In contrast to vertical jump performance, single-leg hop distance was not significantly associated with the corresponding knee torque values. This finding supports the view that hop performance depends on a broader set of neuromuscular and mechanical factors than isolated torque production alone (18, 27). Horizontal hop tasks require coordinated contributions from the hip, knee, and ankle, in addition to dynamic control during take-off and landing. As a result, athletes may achieve comparable hop distances through different mechanical solutions, which may weaken the direct association between hop distance and isolated knee extensor or flexor capacity. This interpretation is also consistent with previous work suggesting that functional hop performance may mask residual deficits when individuals compensate through altered joint contribution or movement strategy (14, 28). From a clinical perspective, these findings suggest that hop tests remain useful for evaluating task performance and movement capacity, but should not be used in isolation to infer adequate knee strength recovery.

### Functional symmetry does not guarantee strength symmetry

The weak association between SYMMETRY_JUMP and SYMMETRY_HOP (*r* = 0.323), combined with the absence of association between functional symmetry and quadriceps strength symmetry, reinforces the idea that different tasks may produce similar inter-limb scores for different physiological reasons. Previous studies have shown that limb symmetry indices derived from functional tests can overestimate recovery by failing to capture residual joint-specific impairments (18, 19). Moreover, proprioceptive adaptations, task familiarization, and movement redistribution across adjacent joints may contribute to apparently symmetrical performance despite persistent deficits in knee torque production (29). In the present study, the lack of association between strength symmetry and functional symmetry suggests that even apparently symmetrical field-based performance may coexist with underlying asymmetries in torque production. This has important implications for clinical interpretation. A high LSI in hop or jump tests may indicate balanced task performance, but not necessarily restoration of muscle function at the knee. For this reason, LSI values should be interpreted cautiously and preferably alongside strength-based or instrumented measures when decisions about rehabilitation progression or return to sport are being made. Although hamstring torque was assessed, hamstring symmetry was not included among the primary symmetry outcomes because the prespecified symmetry analysis focused on quadriceps torque and field-based measures most commonly used in return-to-sport monitoring.

### Independence of mSEBT and hop performance

Although both the mSEBT and the single-leg hop test demonstrated strong inter-limb associations, they were not significantly correlated with one another. This lack of association suggests that the two tests assess distinct aspects of lower-limb function. The mSEBT is more strongly influenced by postural control, sensorimotor integration, trunk stabilization, and the ability to regulate center-of-pressure displacement during a constrained reach task, whereas hop performance places greater demands on rapid force production, horizontal propulsion, impact attenuation, and landing control (18, 30). Thus, combining these tests may provide complementary rather than redundant information. From a practical perspective, this finding is relevant for sports physical therapy and athlete monitoring. If two tests are not strongly associated, using both may improve the breadth of clinical assessment by capturing different movement capacities. This is particularly valuable in contexts in which return-to-sport decisions require a broader understanding of neuromuscular readiness rather than reliance on a single task outcome.

### Integrative interpretation

Taken together, the present findings show that functional tests are clinically useful, but each appears to reflect different neuromuscular attributes. Vertical jump performance showed some association with quadriceps torque, whereas single-leg hop and mSEBT performance appeared to reflect more task-specific components such as coordination, dynamic control, and movement strategy. In addition, functional symmetry did not parallel strength symmetry. These results support the use of a multimodal assessment framework in which field-based tests are interpreted alongside objective strength measures, rather than as substitutes for them. Such an approach may improve clinical reasoning when screening athletes, monitoring rehabilitation progress, and supporting return-to-sport decisions. In professional soccer, where performance depends on the integration of strength, balance, power, and unilateral control, a combination of complementary tests may be more informative than any isolated metric (31, 32).

### Limitations

This study has limitations that should be considered when interpreting the findings. First, only male professional soccer players were included, which limits generalizability to female athletes, youth players, or recreational populations. Second, the cross-sectional design precludes causal inference and does not allow the present associations to be interpreted as predictors of injury risk or future performance. Third, isokinetic testing was performed only at 60 °/s and under concentric conditions, which emphasizes maximal torque production but does not capture high-velocity or eccentric muscle function that may also be relevant in soccer. Fourth, the absence of electromyographic, kinematic, or kinetic data prevents a more detailed understanding of the compensatory strategies underlying task performance. Fifth, the analytical approach was based on pairwise correlations only. This was appropriate for an initial construct-level examination of how commonly used functional tests relate to isolated knee torque, but it does not allow adjustment for potential confounders such as body mass, recent training exposure, or playing position, nor does it quantify the relative contribution of multiple strength-related factors within a multivariable framework. Sixth, torque was analyzed in absolute units rather than normalized to body mass. This decision preserved the direct interpretation of the dynamometer output within a relatively homogeneous professional cohort, but normalized analyses may provide complementary insight in future studies. Finally, because complete isokinetic data were not available for all athletes, some analyses involving torque were conducted on a slightly smaller subsample.

### Future directions

Future studies should use longitudinal designs to examine whether combining functional and strength-based measures improves the identification of athletes at risk of underperformance, incomplete recovery, or reinjury. Expanding the analysis to different sexes, competitive levels, and age groups would also improve external validity. In addition, integrating motion analysis, force platform data, and electromyography may help clarify which neuromechanical factors explain the divergence between functional test performance and isolated torque measures. Studies with larger samples should also examine normalized torque variables, hamstring symmetry, and multivariable models that account for potentially relevant covariates. This may ultimately support the development of more precise and individualized criteria for rehabilitation progression and return to sport.

### Clinical implications

From a clinical perspective, the present findings indicate that the mSEBT, hop tests, and vertical jump tasks should be viewed as complementary rather than interchangeable tools. In return-to-sport contexts, apparently symmetrical hop or balance performance should not be interpreted as confirmation of full restoration of knee muscle function; pairing field-based tests with objective strength assessment may reduce the risk of overlooking residual deficits. In routine performance monitoring, bilateral and unilateral jump tasks may provide a practical overview of explosive output, whereas the mSEBT and hop tests may help characterize unilateral control, dynamic balance, and task-specific movement capacity. Accordingly, integrating objective strength assessment with field-based functional testing may improve the detection of residual deficits, support more individualized rehabilitation planning, and strengthen return-to-sport decision-making.

## Conclusion

In professional male soccer players, functional tests such as the mSEBT, hop tests, and vertical jump tasks appear to capture complementary rather than interchangeable aspects of lower-limb performance. While bilateral vertical jump performance was moderately associated with quadriceps torque, mSEBT and single-leg hop performance were not related to isolated knee strength, and functional symmetry did not mirror strength symmetry. These findings should be interpreted as task-specific relationships rather than predictive effects. Accordingly, combining functional tests with objective strength assessment may provide a more comprehensive basis for athlete monitoring, rehabilitation, and return-to-sport decision-making.

## Data Availability

The raw data supporting the conclusions of this article will be made available by the authors, without undue reservation.
